# How to Live in the Moment: The Methodology and Limitations of Evolutionary Research on Consciousness

**DOI:** 10.1111/cogs.70053

**Published:** 2025-03-19

**Authors:** Christian R. de Weerd, Leonard Dung

**Affiliations:** ^1^ Centre for Philosophy and AI Research (PAIR) University of Erlangen‐Nürnberg (FAU); ^2^ Institute of Philosophy II Ruhr‐University Bochum

**Keywords:** Animal consciousness, Evolution of consciousness, Evidence of consciousness, Phenomenal consciousness, Nonhuman consciousness

## Abstract

There is much interest in investigating the *evolution question*: How did consciousness evolve? In this paper, we evaluate the role that evolutionary considerations can play in *justifying* (i.e., confirming or falsifying) hypotheses about the origin, nature, and function of consciousness. Specifically, we argue against what we call *evolution‐first approaches to consciousness*, according to which evolutionary considerations provide the primary and foundational lens through which we should assess hypotheses about the nature, function, or distribution of consciousness. Based on the example of Walter Veit's account and additional reasoning, we contend that evolution‐first approaches struggle to provide compelling empirical evidence for their key claims about consciousness. In contrast with these approaches, we argue that consciousness science needs to foundationally rely on experimental and observational evidence from humans and other present‐day animals. If our arguments succeed, then researchers, when investigating consciousness, are better advised to take as their primary source of evidence consciousness’ present, not its past. Having said this, we acknowledge that evolutionary thinking plays an important role in consciousness science. We delineate this role by stressing several ways in which evolutionary considerations can substantially help advance consciousness research, although in a manner that avoids the evolution‐first approach. Since our argument only concerns the assessment of hypotheses (the “context of justification”), it leaves it open which role evolutionary considerations play in generating hypotheses (the “context of discovery”). That is, evolutionary considerations may nevertheless play a foundational role in hypothesis generation in consciousness science.

## 1 Introduction

Phenomenal consciousness (henceforth: consciousness) is the subjective character of experience: what it is like to be a particular organism, or what it is like to be in a particular mental state (Block, 1995; Nagel, 1974).^1^ Like all biological traits, consciousness has an evolutionary history. For the sake of the argument, we will also assume that it has an *evolutionary function*.^2^ The evolutionary function of consciousness consists of the fitness‐increasing effects consciousness has been selected for by natural selection (Neander, 1991).^3^ This assumption entails that consciousness plays a causal role. This causal role we will call consciousness’ *psychological function*, where “psychological” is understood in a sense which is entirely neutral regarding what the causal role of consciousness is. We may, for instance, understand this causal role in terms of contributions to the complex capacities of the overall cognitive system containing it (Cummins, 1975) but our argument does not depend on it. A central research target in consciousness science, on which no agreement among leading researchers has been reached (Birch, 2022; Irvine, 2012; Schwitzgebel, 2020; Seth & Bayne, 2022), is a detailed and general theory of the nature, biological substrate (e.g., relevant neural areas), psychological function, and evolutionary function of consciousness. Relatedly, there is currently no full and agreed‐upon account of the *distribution question*: Which organisms are conscious?

We adopt the minimal assumption that scientific inquiry can at least contribute to our understanding of the nature, distribution, and function of the physical or functional properties associated with consciousness (Chalmers, 2010) and our aim is to examine how this inquiry is most fruitfully conducted. This scientific project can be fruitfully developed across the metaphysical board, whether dualism or physicalism is true (Chalmers, 2010, p. 40).^4^ Thus, we remain neutral to what *extent* identifying the physical/functional basis of consciousness solves the hard problem (Chalmers, 1996), with the answer largely depending on one's metaphysical views on consciousness (see Chalmers, 2003).^5^ Accordingly, our arguments against the evolution‐first approach (described below) are meant to question whether the approach fruitfully contributes to identifying the (distribution and origin of) physical/functional properties associated with consciousness.

Many researchers have provided extensive and influential accounts that aim to answer the *evolution question*: how did consciousness evolve (e.g., Dennett, 2017; Ginsburg & Jablonka, 2019; Godfrey‐Smith, 2016a, 2020c; Feinberg & Mallatt, 2016; Veit, 2023b)? In this paper, we challenge a particular approach that has been used to answer this question, and questions about the nature, function, and distribution of consciousness in general, which we call *evolution‐first*. This approach says that evolutionary considerations generate independent and foundational evidence to assess hypotheses about the nature, functions, or the distribution of consciousness. If our arguments are successful, then researchers, when addressing the evolution question, are better advised to take as the primary source of evidence consciousness’ present, not its past. Having said that, we do not claim that researchers should eschew evolutionary thinking altogether. Instead, we will outline how evolutionary thinking can play a secondary, but important, role in consciousness science.

The paper is structured as follows. In Section 2, we describe the evolution‐first approach to consciousness and give some reasons why such an approach is *prima facie* appealing. Afterward, we will contextualize this approach within overall scientific methodologies and describe a body of work by Walter Veit, which we take to be the most‐developed and compelling example of the evolution‐first approach. In Section 3, we expose some weaknesses of Veit's account and argue more generally that other evolution‐first approaches may fare no better. In Section 4, we will explore the implications of our argument for other prominent accounts of the evolution of consciousness, specifically Ginsburg & Jablonka's and Godfrey‐Smith's. In Section 5, we will delineate in what ways evolutionary thinking, in a secondary role, can help advance consciousness research.

## 2 Evolution‐first approaches to consciousness

### 2.1 The appeal of evolution‐first approaches

The appeal of evolutionary approaches to consciousness can be brought out by considering the challenges other approaches face. Most approaches to the nature and distribution of consciousness draw on insights from human consciousness in one way or another. The early science of consciousness primarily focused on identifying correlations between neural states and conscious states in humans, where the presence of conscious states is inferred from the subject's verbal reports (Crick & Koch, 1998; 2003). Contemporary consciousness science attempts to move beyond stating mere correlations and postulates theories that explain them (Seth & Bayne, 2022), but its primary evidentiary basis remains human‐centered.^6^

The science of *animal* consciousness, a young and rapidly developing field that deals with the distribution question, has also retained anthropocentric ties. *Theory‐heavy* approaches first provide a theory of human consciousness and only afterward examine whether nonhuman systems satisfy the criteria spelled out by the theory (e.g., de Weerd, 2024; Tononi & Koch, 2015). *Theory‐light* approaches commit to a broad hypothesis on the relation between cognition and consciousness that is compatible with a wide range of theories of human consciousness (e.g., Birch, 2022). *Theory‐neutral* approaches aim to further reduce the dependence on theoretical assumptions but still typically argue from analogy between humans and nonhuman systems in terms of structural or functional similarity (Dung, 2022).^7^ Relatedly, many researchers maintain that even those approaches to nonhuman consciousness which deliberately try to steer away from, or go beyond, anthropocentrism must use human consciousness as their starting point (see, e.g., Andrews, 2024; Dung & Newen, 2023; Suzuki, 2022, p. 422; McKilliam, 2024, p. 9).

However, any human‐centered approach brings along challenges. The sole usage of human evidence limits both the amount and diversity of available empirical information. Moreover, consciousness might be implemented by different (neural) mechanisms in nonhuman animals than in humans (Godfrey‐Smith, 2016b). Additionally, it might be that contemporary theories of human consciousness, or approaches tied to human consciousness in general, track features or dimensions that are not necessary for consciousness (Veit, 2022a), in which case there is an insensitivity to simpler forms of consciousness that lack these features or dimensions. Although they may be surmountable, the challenges presented by anthropocentrism make it tempting to opt for an approach that is not human‐centered. Moreover, it is often fruitful to describe and explain systems from the design stance (Dennett, 1987); that is, in terms of what they have been designed to do. For these reasons, approaching questions about consciousness from an evolutionary angle seems to offer an attractive alternative.

Everyone is likely to agree, in principle, that the evolution question is worthwhile and scientifically interesting. It includes fascinating questions such as when consciousness first arose, when different forms of consciousness developed in the evolutionary tree of life, which adaptive pressures and biological constraints drove this development (involving consciousness’ evolutionary function), and how the evolution of consciousness interacted with the evolution of other cognitive processes. However, evolution‐first approaches go beyond simply asking the evolution question; they propose a certain *methodology* for studying consciousness. Specifically, they are characterized by a certain view of the priority, explanatory power, and independence of the evolution question and of evolutionary considerations in general.

According to the other, predominant, view, consciousness science should start with human consciousness and the distribution question. Afterward, the resulting accounts of human and nonhuman consciousness can be used to evidentially support hypotheses about the evolution question. On this picture, assessments of the evolution question are heavily constrained by (synchronic) human and animal consciousness research. As such, evidence about the evolution of consciousness consists, primarily and foundationally, of evidence about the present nature, psychological functioning, and distribution of consciousness. On this view, evolutionary thinking plays a secondary role in our understanding of the nature and function of consciousness. It supplements evidence derived from current human and animal consciousness research, but it cannot provide the central source of evidence for supporting claims about consciousness.^8^ We endorse this view.

Evolution‐first approaches, by contrast, hold that the primary and foundational lens through which we should assess hypotheses about consciousness is in virtue of evolutionary considerations. That is, in evolution‐first approaches, evolutionary considerations generate *independent* and foundational evidence on the nature, psychological function, and distribution of consciousness. So, on this view, evidence derived from evolutionary reasoning and an evolutionary context heavily constrains testing of our views of the nature, psychological function, and distribution of consciousness. This means that the chain of evidence is not top‐down—from contemporary evidence about consciousness to the evolution question—but bottom‐up, in that the primary evidentiary basis that should be used to assess our hypotheses on what consciousness is in the present is primarily grounded in an evolutionary context and evolutionary reasoning.

This bottom‐up reasoning requires that there are some sources of evidence on the evolution question which are independent from what can be learned by studying consciousness in living animals. This is because our views on the evolution question cannot lend additional support to our views on consciousness in the present if our views on the evolution of consciousness are relatively straightforwardly derived from views about consciousness’ present. For example, one cannot posit a certain theory of consciousness, subsequently note that this theory implies (in conjunction with some accepted facts about animal evolution) that consciousness first arrived at time *t* over the course of animal evolution, and then take the correspondence between the theory and one's view that consciousness arrived at time *t* as further evidence for the theory. This chain of reasoning would be *viciously* circular. By contrast, as soon as there is independent evidence for the view that consciousness arrived at *t*, this does provide positive evidence for a theory with that prediction. Similarly, if a theory's prediction does not correspond to independent evidence for the view that consciousness arrived at *t*, such evidence would constitute defeasible evidence *against* such a theory, or at the very least be suggestive that the theory needs to be amended. Moreover, any independent evidence that evolutionary considerations provide would need to be foundational; that is, needs to be powerful enough to serve as a central basis of consciousness science.^9^

In other words, evolution‐first approaches reject limiting oneself to making inferences top‐down based on knowledge about which properties consciousness currently has to its evolution. Instead, these approaches assume that (1) evolutionary considerations provide us with independent and foundational evidence on the evolution question (2) such that evidence on the evolution question in turn provides us with knowledge about consciousness’ present properties or distribution. Since 1 is the central point of contention, we will largely focus on it in what follows.

Note that evolution‐first approaches can grant that evidence of consciousness’ present should play a crucial role in consciousness science. Their claim is only that evolutionary considerations should play a foundational role in assessing hypotheses in consciousness science which is not dependent on a prior, independent level of theorizing and experimentation. Thus, they can admit that contemporary evidence is indispensable. In the next subsection, we will contextualize the evolution first‐approach and our argument with respect to general questions of scientific methodology.

### 2.2 Background scientific methodologies

First, we adopt the well‐established distinction between the context of justification and the context of discovery in science (Hoyningen‐Huene, 1987; Schickore, 2022, sect. 5), which is commonly understood as follows. The context of justification is about the testing and rational assessment of scientific hypotheses: how they can be supported and evaluated in recourse to evidence and argument. By contrast, the context of discovery is about the generation of new hypotheses and ideas. Our arguments against the evolution‐first approach are focused only on the context of justification, as the evolution‐first approach—as we defined it—only makes claims about the testing and assessment of hypotheses, not their generation.^10^

In the context of justification, we can distinguish confirmatory and falsificationist methodologies. According to confirmatory methodologies, science should search for evidence which positively justifies a hypothesis or makes it likely that a hypothesis is true. Hypothetico‐deductivism and Bayesianism are both examples of confirmatory methodologies (Crupi, 2021). According to falsificationist methodologies (Popper, 1959; Stewart, 2022), scientific hypotheses can never be positively confirmed or justified. Instead, they can only withstand an increasing number of tests aimed at falsifying the hypothesis. However, no matter how many falsificatory tests a hypothesis survives, it always remains “provisional, conjectural, hypothetical” (Thornton, 2022, italics removed).

Our characterization of the evolution‐first approach and our arguments below are mostly framed in a confirmatory way. This is because the authors we respond to use this idiom: they mostly talk about supporting and finding evidence *for* hypotheses, rather than attempting to falsify them. However, since our arguments are quite general, they can be cast in ways adhering to confirmatory or falsificationist methodologies, respectively. On a confirmatory reading, evolution‐first approaches assume that evolutionary considerations provide independent and foundational evidence for certain views on the evolution question. On a falsificationist reading of these approaches, instead, they claim that such considerations can be used as independent and foundational tests to potentially falsify some hypotheses.

Generally, when we discuss below whether some piece of evidence E confirms some hypothesis H, a falsificationist can instead ask themselves whether Not‐E falsifies H. Our arguments generalize to both types of questions (we will give examples below). Importantly, at least in cognitive science (Woensdregt et al., 2024), and specifically in consciousness science (McKilliam, 2024), it is not plausible that a single piece of evidence can decisively confirm or falsify a theory. Thus, we assume that, on both views, hypothesis testing is a gradual affair in which evidence needs to accumulate over time (Corcoran, Hohwy, & Friston, 2023; Melloni, 2022).^11^

In the next subsection, we will present one example of the evolution‐first approach in detail.

### 2.3 Veit's evolutionary approach

We regard a recent body of work by Walter Veit (see, e.g., 2022a; 2022b; 2023a; 2023b) as the most‐developed and compelling example of an evolution‐first approach to consciousness. So, we will first describe and discuss Veit's view and then argue that our arguments generalize to all evolution‐first approaches. Veit confirms that his view is an “evolutionary bottom‐up approach to consciousness” (Veit, 2023b, p. 113), according to which we ought to “force the problems of consciousness through th[e] theoretical bottleneck of evolutionary theory” (Veit, 2023b, p. 116). This view is claimed to promote a Copernican shift from a human‐centered field toward a “true Darwinian science of consciousness in which evolutionary origin, function, and phylogenetic diversity are moved from the field's periphery to its very center” (Veit, 2023b, p. 1). Relatedly, Veit posits that to “reconstruct the possible evolutionary origins and function of consciousness, we are engaged in the paleobiologist's effort of making sense of a trait by connecting its extant ‘users’ with its historical traces” (Veit, 2023a, p. 177).

This suggests, then, that Veit does not just want to suggest that evolutionary considerations merely *inspire* a tentative hypothesis about the possible nature of consciousness, in the form of the pathological complexity thesis (PCT) (context of discovery), but instead suggests that evolutionary considerations can vindicate the PCT itself (context of justification). As Veit puts it, “by making sense of the lifestyle changes of animals preceding the Cambrian explosion, we will be able to *explain* the dawn of subjective experience in the form of a basic feel of evaluation” (Veit, 2023b, p. 22, emphasis added; see also Section 3). Evolutionary considerations are supposed to take the center stage and serve as the primary evidentiary basis to assess which properties consciousness has in the present—its nature and structure, psychological function, and distribution—by elucidating its phylogenetic history and its evolutionary function.^12^

Veit's evolution‐first approach leads him to the following view. First, he identifies a specific evolutionary transition event as important for the origins of consciousness. Specifically, Veit observes that during the Cambrian explosion of animal diversity 540−520 million years ago (mya), the “pathological complexity” (PC) of many animals increased significantly. PC, roughly, is the trade‐off problem between competing actions that organisms could take when faced with challenges and opportunities to maximize their fitness during their lifecycles (Veit, 2023b); for example, whether to depress or enhance immune responses to external changes, or choosing the best course of action to steal an unsupervised fledgling from a bird's nest (Veit, 2023a).^13^ Veit takes PC to be a universal obstacle to life but maintains also that the Cambrian explosion introduced more variety, and a large increase, of complex biological organizations with high degrees of behavioral flexibility. This greatly exacerbated the economic trade‐off problem of how to coordinate these new degrees of freedom.

The increase in PC during the Cambrian explosion, then, is taken as the problem that organisms needed to solve to maximize fitness. It is here that Veit takes consciousness to provide a solution as to how to coordinate these new degrees of freedom. Specifically, Veit argues for his so‐called^14^:

**Pathological complexity thesis**: The function of consciousness is to enable the agent to respond to pathological complexity (Veit, 2023a, p. 175).

Veit claims that the solution to the problem of PC requires an evaluative system that utilizes a common currency to enable efficient, economic, trade‐off decisions between a wide array of available actions (also see Cabanac, 1992; 1996; Grinde, 2023). According to Veit, the property that plays the role of this common currency is *hedonic* or *valenced consciousness*; conscious states that feel good or bad (Veit, 2023a, p. 176). In doing so, organisms utilize a hedonic evaluative system that allows them to make trade‐off choices based on a common scale of hedonic value. The choice that invokes the most positive feeling (or least negative) is the choice that is made and acted on. The common currency of hedonic consciousness, then, facilitates the action selection mechanism that ultimately is an adaptive response to PC. The evolution‐first approach to consciousness, then, is at full display here. The Cambrian explosion *reveals* a problem that consciousness is taken *plausibly* to solve (Veit, 2023a, p. 205). In accordance with this account, Veit argues that hedonic consciousness is the most basic form of consciousness out of which other dimensions of consciousness were subsequently built (Veit, 2022a). The main subsequent dimension is *sensory consciousness* of one's external and internal environments. This is the perceptual consciousness of what one sees, smells, touches, and tastes. In both cases, Veit appears to provide *arguments* for believing his views: for the PCT and for the view that hedonic consciousness is the fundamental dimension of consciousness. This entails that we are operating here in the context of justification.

For Veit, then, the evolution question comes prior to, or is at least as foundational as, the distribution question. Reasoning about the putative evolutionary history of consciousness supports claims about the evolutionary function of consciousness, which—it is claimed—can then inform predictions about the presence of consciousness in other animals (Veit, 2023b, p. 2). Because this assumes a significant degree of independence of evolutionary considerations from knowledge of human and animal consciousness gained via other sources, and evolutionary considerations are taken to be foundational in assessing the PCT, this makes it an evolution‐first approach in our sense.

Moreover, evolutionary reasoning is also taken to *justify* decisions between different views of the origin of consciousness. For example, one view says consciousness evolved near the start of the Cambrian times (∼540 mya) but another allows that it evolved earlier, in the Avalon times around 575 mya (Veit, 2023a; also see Erwin & Valentine, 2013). An especially influential advocate of the Cambrian view is Godfrey‐Smith (2016a; 2016b: 2019; 2020a; 2020b; 2020c), who ties the origin of conscious experience to the appearance of complex evaluative, sensory, and motor capacities of the first vertebrates, arthropods, and so on.^15^ However, Veit points out that some animals—which include certain worms and slug‐like creeping mollusks—evolved complex sensorimotor capacities during the previous Avalon explosion but nevertheless went extinct. Veit takes his PCT to provide a natural explanation of this event, and thus to be confirmed by it. According to Veit, despite animals from both the Avalon and Cambrian explosions exhibiting complex sensorimotor capacities, animals from the Avalon event lacked, whereas organisms from the Cambrian one had, the evaluative system required to deal with PC (Veit, 2022b, p. 175). In the next section, we will present arguments against Veit's view in particular and evolution‐first approaches in general.

## 3 Resisting the evolution‐first approach

### 3.1 Weaknesses of Veit's approach

According to our reconstruction, Veit's account has two central, interrelated, tenets. First, hedonic consciousness is the most fundamental dimension of consciousness. Second, the function of consciousness is dealing efficiently with PC. We will expose a dilemma here: either the evidence he uses to support these tenets is independent of evolutionary reasoning, and thus does not conform to the evolution‐first approach, or else the arguments are based on evolutionary considerations which are unconvincing. While we cannot engage with all aspects of Veit's rich account, we pick the aspects which serve best to illustrate our general concerns.

#### 3.1.1 Hedonic consciousness as fundamental

Starting with the first tenet, Veit's claim that hedonic consciousness is fundamental relies on an argument by elimination: he considers the five dimensions of consciousness postulated by Birch, Ginsburg, and Jablonka (2020) and rules out the other four dimensions as candidates. We will illustrate our dilemma by focusing on his arguments that the dimensions of self‐consciousness and synchronic unity, respectively, are not fundamental.^16^

Veit's argument that self‐consciousness is not fundamental to consciousness exemplifies an evolution‐first approach but is not compelling. It is based on the claim that consciousness does not necessarily involve self‐consciousness. First, he argues against the autopoietic view of cognition (Thompson, 2010) and the misunderstanding that mere self‐registration, arguably necessary for all cognition, entails self‐consciousness.

His first *positive* argument for ruling out self‐consciousness appears to be that self‐consciousness cannot be assembled gradually, which raises questions about how it could have evolved at all. This argument fits well with an evolution‐first approach, because its central premise is a constraint based on evolutionary reasoning. Here is the argument in full: Given the evolutionary assumption that consciousness gradually acquired more sophistication, it follows that, if a dimension of consciousness (self‐consciousness here) is taken to be fundamental to consciousness *as such*, then it should be amenable to a gradualist account; ergo, self‐consciousness is not fundamental. So far so good, but we reject the initial claim that self‐consciousness could not have evolved gradually. Our central concern is that even Birch et al. (2020), on which Veit relies, explicitly view self‐consciousness as graded and even distinguish three degrees of it: 1. Registering some experiences as representing internal bodily events and other experiences as representing events in an external world, 2. Awareness of one's own body as a persisting object that exists in the world, and 3. Awareness of oneself as the persisting subject of a stream of experiences, distinct from other such subjects.

Veit's remaining argument is, as we understand it, that it is unclear what evolutionary function consciousness could have if it fundamentally involves self‐consciousness. That is, it is not clear how there could be any adaptive role or evolutionary selective pressure for refined forms of self‐consciousness, so self‐consciousness cannot be fundamental. This contrasts with *sensory* consciousness, which is not challenged by this argument, because it is highly adaptive in the sense that better sensory abilities aid survival (the same for evaluative consciousness).

This second argument also fits with an evolution‐first approach since it is based on reasoning about the evolutionary function of consciousness. Yet, we are not convinced that it is sound. The comparison to sensory consciousness is misleading because the selective pressures for more sophisticated perception may be distinct from the selective pressures for perceptual *consciousness* specifically. For his argument, Veit needs to argue for an evolutionary function of perceptual *consciousness* specifically. He must say what consciousness adds—aside from perception which seems to sometimes occur unconsciously (Michel, 2023; Prinz, 2015)—and explicate its psychological function. Without making assumptions about consciousness’ typical causes and effects, it is impossible to make informed and testable claims about its evolutionary function. However, whatever view he chooses here needs to be grounded in evidence from human and animal consciousness science, not evolutionary hypotheses. So, synchronic evidence about consciousness has to take center stage, not evolutionary reasoning.

Otherwise, speculation about evolutionary functions of consciousness would not be constrained by empirical evidence, so evolutionary stories would be easy to come by. One can easily tell numerous (just‐so) stories where self‐consciousness has an evolutionary function. For instance, here is one view that is diametrically opposed to Veit's view that evaluative consciousness is fundamental: Humphrey (2020, 2022) claims that a key evolutionary function of consciousness is to facilitate understanding of one's own mind, which facilitates possession of a model of the mind which can then be used to predict and explain others’ behavior. It is easy to see why this ability would be adaptive and why this function would be related to self‐consciousness in particular. Thus, if this evolutionary story were true, self‐consciousness would be fundamental.

Note that our objections also hold if Veit's argument is understood from a falsificationist perspective. For example, for the very reasons we point out, the evolutionary assumptions that consciousness evolved gradually and that it has an evolutionary function fail to falsify the view that self‐consciousness is the fundamental dimension of consciousness. Importantly, the view that self‐consciousness is fundamental can be reconciled with opposing evolutionary assumptions, so that they *cannot* falsify it. Thus, these evolutionary considerations also fail as a test of the hypothesis that self‐consciousness is fundamental if we assume the falsificationist methodology.

So, Veit's evolutionary arguments for the view that self‐consciousness is not fundamental are unsuccessful. Moreover, they fail for generalizable reasons: Arguably, for every central aspect of consciousness, it is possible to conceive of it as graded and to tell a plausible‐sounding story about its putative evolutionary function.

Synchronic unity characterizes the extent to which an organism's conscious experience is integrated and unified at any given time. Veit argues that synchronic unity is not necessary for consciousness, and thus not fundamental (Veit, 2022a). For this argument, he relies on empirical evidence from human split‐brain cases (de Haan et al., 2020) and considers that some animals may be “natural” split‐brains (Carls‐Diamante, 2017). It is contested whether these findings indicate a form of disunity of consciousness (Bayne, 2008) and, if so, whether they indicate disunity of *phenomenal* consciousness, as opposed to, for example, access‐consciousness (Bayne & Chalmers, 2003). Nevertheless, it seems to us that this is the kind of evidence which could in principle decide questions about which features are necessary for, and ultimately fundamental to, consciousness. Note, however, that this line of thought is based on contemporary experimental evidence; it is entirely independent of evolutionary reasoning.

These are only examples, but we submit that they illustrate a general pattern: The evidence considered is either based on evolutionary considerations but unsuccessful or independent of an evolution‐first approach.

#### 3.1.2 The pathological complexity thesis

Veit's second tenet is the PCT. The same dilemma applies. For the sake of the argument, we grant both that valence is centrally related to dealing with PC^17^ and that hedonic (valenced) consciousness is fundamental to consciousness. According to Veit, evolutionary considerations generate independent evidence for the PCT that should lead us to accept that the PCT is correct, or at the very least increase our credence in the PCT.^18^

However, an overarching problem, which we take to generalize to the evolution‐first approach as such, is that it is unclear *how* evolutionary considerations can generate independent evidence for a function of *consciousness*, as opposed to some nonconscious mechanism or process. Suppose we grant that an evaluative system, utilizing some kind of common currency, is required to deal with the explosion of PC that occurred during the Cambrian explosion. Arguably, what this establishes, at most, is that dealing with PC is the function of valency, not consciousness. It is left open what function consciousness specifically adds to unconscious valency.^19^

It is worth noting that Veit explicitly concedes that nonconscious means *could* be levied to deal with PC (Veit, 2023a, p. 176; Veit, 2023c, p. 204). However, Veit argues that, through the Cambrian explosion, hedonic consciousness becomes *worth having* (Veit, 2023a, p. 176; Veit, 2023c, p. 204), that is, gains sufficient adaptive value. The reason is that, according to Veit, dealing with PC in terms of an evaluative system that *specifically* utilizes hedonic consciousness as its common‐currency is more efficient, and is likely easier to achieve compared to nonconscious alternatives (Veit, 2023c, p. 205). Thus, despite the availability of nonconscious alternatives, Veit considers the need for an evaluative system during the Cambrian explosion to constitute evidence, albeit defeasible, in favor of the PCT.

We do not see how this reply can work. First, the claim that hedonic consciousness becomes worth having when PC increases during the Cambrian explosion already assumes that it is, or can be, a (psychological or evolutionary) function of hedonic consciousness to deal with PC. But to assume this is simply to presuppose that the PCT is (potentially) correct. Moreover, Veit does not give evidence that dealing with PC using evaluative consciousness should be more efficient and easier than alternatives. To support such a claim, one would need to base it on theoretical assumptions about consciousness that go well beyond the evolutionary considerations that Veit considers. As such, the need for a specific evaluative system during the Cambrian explosion does *not* constitute evidence that confirms the PCT.

To be clear, our argument does not rest on the general view that because consciousness is not defined in functional terms, all behavior could be explained without invoking consciousness (this view is connected to the “explanatory gap” (Levine, 1983)). Instead, we have assumed that consciousness has a function and that empirical evidence for such a function may *in principle* be provided. The point is, rather, that Veit's argument suggests that PC is involved in the function of valence, but there is no reason given why PC should be connected to consciousness, rather than valence. Such evidence could be given in principle. For instance, if a brain mechanism which has special relevance for dealing with PC is differentially active when stimuli are perceived consciously versus unconsciously (as measured, e.g., by verbal report). However, it is not clear how evolution‐first approaches can provide compelling evidence here (or how they could form the basis of a strong falsification attempt). So, our argument is based on concrete empirical assumptions, rather than broad metaphysical ones.

We are neutral on the claim that valence is at the core of consciousness and its origin. We are merely claiming that behavioral and neuroscientific evidence from contemporary animals (including humans) will play the foundational role in answering these questions, not evolutionary considerations along the lines Veit suggests. Similarly, we are open to the possibility that the PCT may be established via an inference to the best explanation which relies primarily on a wide variety of experimental evidence from contemporary animals, supplemented by evolutionary considerations. But focusing on an evolutionary hypothesis is not sufficient for being an evolution‐first approach. Instead, the kind of evidence and arguments appealed to in favor of the hypothesis are central.

So far, we have argued that it is unclear how the PCT's evolutionary considerations can constitute independent evidence *in favor* of a hypothesis about the function of consciousness. However, it is equally unclear how evolutionary considerations can count *against*, or falsify, such hypotheses. Consider, for instance, the hypothesis of phylogenetic splits between sensory and evaluative capacities in organisms (Godfrey‐Smith, 2020a). Such organisms could have evolved, for instance, very rich sensory capacities but very sparse evaluative capacities; call such organisms *sensory‐specialists*.^20^ On the assumption that complex capacities are associated with consciousness, whereas simple capacities are not, sensory‐specialists would only have sensory, but no evaluative, consciousness.^21^

According to Veit, if sensory‐specialists (were to have) exist(ed), they (would) constitute direct evidence against, and thus potentially falsify, the PCT, because the PCT does not allow for stand‐alone sensory consciousness.^22^

However, it is not clear why the existence of sensory‐specialists is supposed to falsify the PCT. Simply put, Veit's sensory specialists could have had complex but *nonconscious* sensory processing in addition to lacking evaluative consciousness. Such entirely nonconscious sensory specialists would not offer any evidence at all about a hedonic function of consciousness—neither *against*, as Veit claimed, nor for it.^23^ The issue is that Veit's argument presupposes that sensory specialists would be conscious, but no evidence has been provided (and Veit's theory even suggests the opposite). This, again, suggests that the evolution‐first approach also fails to falsify hypotheses about consciousness. Since relevant evidence could be provided in principle, for example, by testing the learning abilities of such sensory specialists (Birch, 2022), this is not an instance of the general explanatory gap worry about consciousness.

What about Veit's suggestion that the PCT can be fruitful for consciousness research? He claims that by using the PCT “we are placed in a better position to make predictions regarding [animals’] subjective experiences, which can then be used in a feedback process to better understand their pathological complexity, thus ultimately allowing us to create an evolutionary framework for the study of animal consciousness” (Veit, 2022a, p. 187). Perhaps, then, starting with a tentative hypothesis about the function of consciousness is required to make any meaningful progress. However, this kind of reasoning can also be adduced in favor of any and every competing proposal. As such, without having good empirical reasons to think that the PCT is at least somewhat on the right track, we might as well presuppose any other account of consciousness. Since neuroscientific theories are amenable to experimental evidence, they seem like a less arbitrary starting point.

Our arguments do not imply, of course, that the PCT is false or has no place in consciousness research. The PCT is a perfectly legitimate, though speculative, scientific hypothesis inspired by evolutionary considerations (context of discovery) that can subsequently be assessed in light of available, or future, empirical evidence (context of justification). However, the primary evidence for assessing the PCT will be nonevolutionary, based on experiments and observations of contemporary humans and other animals.

Hence, we do not claim to have refuted Veit's account. However, our objections are intended to put pressure on the distinctive evolutionary standpoint his account adopts. There are major challenges to explaining how genuine evolutionary reasoning, when taken to be foundational, can make progress in consciousness science.

### 3.2 Evolution‐first approaches in general: Are there any possible sources of independent evolutionary evidence?

As we have said, Veit's account seems to us to be the best example of an explicit, systematic, and well‐developed implementation of the evolution‐first approach. This suggests that the dilemma identified above may not be specific to Veit's account, but generalize to many, or all, evolution‐first approaches. To further support this view, we will first provide a general characterization of the evolution‐first approach, and formulate more general challenges afterward.

First, it is necessary to characterize the general features that all evolution‐first approaches share. We already said that (i) their *defining* feature is that they ascribe a foundational and independent role to evolutionary considerations in assessing hypotheses about consciousness (Section 2.1). Such approaches will typically (or always) have certain further, closely related features which provide a more substantive characterization^24^:

(ii) They are based on a specific model derived from evolutionary *theory*.
(iii) They assign an evolutionary function to consciousness.
(iv) Hypotheses about the properties consciousness needs to have to play this evolutionary function play a central role in their arguments.
(v) Historical evidence from the fossil record plays a central role in their arguments.
(vi) They are skeptical of many current influential neuroscientific theories of consciousness.
(vii) They typically stress that we should start our investigations with simple—instead of complex—forms of consciousness.

Many other features of Veit's accounts are not essential for an evolution‐first approach, thus characterized. An evolution‐first approach need not assume that evaluative consciousness is foundational. Instead, other versions of the approach could claim that some other dimension of consciousness came first or that multiple aspects of consciousness appeared simultaneously. Nor must an evolution‐first approach say that consciousness originated during the Cambrian explosion. Other possible times are 3.8 billion years ago in the first living cells (Reber, Miller, Slijepcevic, & Baluška, 2023), in the first reptile‐like amniotes in the later Paleozoic Era (Cabanac, Cabanac, & Parent, 2009), in the Mesozoic Era in the first birds and mammals (Butler, Manger, Lindahl, & Arhem, 2005), or possibly even only in the first humans (LeDoux, 2023).

So, while Veit's account constitutes a paradigmatic example of the evolution‐first approach, this general characterization suggests that other accounts are also candidates for counting as using an evolution‐first approach. Dennett (2017) and Humphrey (2012; 2022) tell different, wide‐ranging natural histories of mind and consciousness. It is notable that they either do not presuppose an explicit theory of consciousness or that their historical account seems to proceed relatively independently of this theory, while they make use of explicit evolutionary assumptions (such as gradualism) when reasoning about consciousness. Merker (2005) identifies a selection pressure related to decision‐making faced by mobile animals, namely, the need to disentangle “target‐related guidance of behavior from movement‐produced contamination of sensory information as well as from the complexities of muscular control” (*ibid*, p. 108). He suggests that consciousness, by providing a simplified “integrated reality space,” was an evolutionary adaptation to solve this problem. Newen and Montemayor (2023) regard as a foundational criterion of adequacy for theories of consciousness—crucial for motivating their ALARM theory—whether the theories ascribe a plausible, survival‐relevant evolutionary function to consciousness. Baluška and Reber (2019, p. 2) argue for their cellular theory of consciousness via an “evolutionarily bottom‐up theory of sentience and consciousness.” Grinde (2023, 2024) develops a model of the evolution of consciousness which is not based on a particular theory and heavily informed by evolutionary reasoning. Grinde claims that consciousness (only) “makes sense in light of evolution” (Grinde, 2024).

While there is a sense in which candidates for evolution‐first approaches in consciousness science abound, we are not aware of previous detailed methodological accounts of the role evolutionary evidence should play in consciousness science. As a result, talk of evolution in consciousness science is often ambiguous between the evolution‐first and more moderate (see Sections 4 and 5) evolutionary approaches. Relatedly, some accounts are only partial evolution‐first approaches, if only a part of the view is independently motivated using evolutionary considerations.

It would require a detailed exposition of the cited accounts to examine satisfactorily whether they are best classified as full evolution‐first approaches; like the one we provided for Veit's account. Instead, we take it to be more fruitful to invite researchers working on consciousness to think carefully, given the tools that we have provided, about the role evolutionary considerations should play in their accounts. If they seek to develop their account as evolution‐first, then they need to contend with the challenges developed in this paper.

One challenge to evolution‐first approaches in general is that they struggle to point to credible *sources* of evidence on the evolution question, or on important features of consciousness. This difficulty arises because the distinctive characteristic of the evolution‐first approach, as defined previously, is that it primarily appeals to features which are not based on contemporary evidence, since this evidence does not itself rely on consideration of the evolutionary history of consciousness. So, if an approach is primarily based on evidence from observations or experiments on living animals, it is not evolution‐first, even if evolutionary considerations appear at a later stage of the approach.^25^ By contrast, the traditional, top‐down, approach to consciousness can readily inform the evolution question because this approach embraces the empirical evidence from the start—using data on consciousness in living animals, on its nature, and on its associated psychological properties. In the traditional approach, for example, evidence of consciousness in living invertebrates is evidence for an early evolutionary origin of consciousness, while evidence that consciousness requires cerebral‐cortical processes is evidence that consciousness is an evolutionary latecomer (see Section 5 for more examples of the important role of evolutionary reasoning in the traditional approach).

The evolution‐first approach can use the fossil record to support its claim that it is the best method for studying consciousness, and it could make the same claim about the archaeological record of human artifacts. But do these historical sources provide the *independent* evolutionary evidence that is required for the approach to be valid? First, let us consider the two sources. From the fossil record, ancient braincases can inform the evolution of consciousness, by documenting enlargements in brain regions that tell when conscious capacities may have advanced, for example, in the first birds about 165 mya (Alonso, Milner, Ketcham, Cookson, & Rowe, 2004). The archaeological record can provide us with evidence about the evolution of psychological traits related to human consciousness. For instance, the increasing complexity of material artifacts such as tools can indicate the emergence of increasingly complex psychological capacities (Overmann & Coolidge, 2019), because manufacturing and learning to use the former (e.g., bifacial hand axes) may require the latter (e.g., a propositional level of reasoning) (see, e.g., Gibson & Ingold, 1993).

Another argument apparently favoring the evolution‐first approach is that it incorporates adaptationist reasoning; that is, thinking about the selective advantages a trait may have brought. Such reasoning is widely considered to play an important role in biology (Olson & Arroyo‐Santos, 2015), comparative cognition research (Halina, 2023), and sometimes in the philosophy of mind (Sterelny, 2003).

Yet, all three of these sources of evidence emphasized by the evolution‐first approach—fossil‐based, archaeology‐based, and adaptationist—require some antecedent assumptions about the psychological function of consciousness which need to be tested via experimental research on present‐day animals. Brain fossils (de Sousa et al., 2023) rely on assumptions about the connection between brain size or structure and consciousness. Adaptationist reasoning relies on antecedent knowledge about the causal role which the capacity in question plays in the larger cognitive system; then, based on assumptions about a capacity's causal role, one can infer how it contributes to behavior and hypothesize how the evolutionary fitness of the organism would be affected if the capacity were not present. Similarly, using archaeology to track consciousness as a psychological trait relies, at least in part, on a contemporary understanding of cognition to make inferences about the evolution of cognitive capacities based on archaeological evidence (*evolutionary cognitive archaeology*: Wynn & Coolidge, 2016, p. 201; Gowlett, 1979; Parker & Gibson, 1979; Wynn, 1979; Davidson, 2010). If one does not make assumptions about the causal role consciousness plays, then one cannot know whether the archaeological artifacts one finds constitute evidence of consciousness.^26^

So, in a nutshell, our argument is based on the view that evolutionary arguments rely on substantive and controversial antecedent assumptions about the psychological function of consciousness. Crucially, this is to a much weaker extent true of contemporary experimental studies on consciousness, which is why our argument does not generalize to them. Many of them rely only on assumptions which are compatible with a wide range of theories of consciousness, for example, that consciousness usually contributes to verbal report or that consciousness facilitates *some* cluster of cognitive abilities (Birch, 2022), to support claims on the psychological function of consciousness. Denying that contemporary evidence can support claims about the psychological function of consciousness would mean claiming that a cognitive science of consciousness, which aims at an account of the psychological function of consciousness, is fundamentally flawed or at least severely limited. We do not have space to sufficiently engage with this radical skeptical view here. However, it is clear that our arguments against evolution‐first approaches do not speak against the view that contemporary evidence can provide knowledge about the psychological function of consciousness and thus do not support this skepticism.

Since our aim here is methodological, we will not argue for a specific theory of consciousness’ psychological function (for a review, see Seth & Bayne 2022). Nevertheless, it seems clear that a compelling theory will mainly be assessed via experimental evidence from humans, particularly in cognitive neuroscience, and other animals and cannot be tested sufficiently via unconstrained evolutionary reasoning.

Of course, the (potential) evolution‐first approaches we discussed could be restricted to the context of discovery (hypothesis generation), while being silent on the assessment of hypotheses. Then, these approaches would be compatible with our argument, but they would also lose a significant part of their ambition.

In the next section, we consider some accounts that combine evolutionary considerations with abundant data from contemporary animals.

## 4 Other views on the evolution question

There are many other prominent accounts which discuss the evolution of consciousness. Initially, these might seem like counterexamples to our argument—evolutionary accounts which are plausible and well‐supported by evidence and strong arguments. However, these accounts do not employ an evolution‐first approach as we have defined it, according to which evolutionary considerations generate independent and foundational evidence on consciousness. Instead, they rely primarily on top‐down methodologies for addressing the evolution question. They appeal to implications of theories of consciousness, draw on evidence on the distribution question, examine cognitive features related to consciousness (e.g., sensory/affective complexity), or some kind of combination of these elements. On these kinds of views, evidence about the evolution of consciousness is primarily based on its present nature, psychological function, and distribution. The viability of such approaches is consistent with our argument because they assign only a secondary role to evolutionary considerations.

We will discuss two notable approaches of this kind, by Ginsburg and Jablonka (2019) and Godfrey‐Smith (2020a; 2020b; see also 2016a; 2020c). Ginsburg and Jablonka's Unlimited Associative Learning framework is based on the following ideas. Whereas there is currently no consensus about the nature or mechanisms of consciousness, researchers might nevertheless agree on a list of capacities that are *jointly sufficient* for consciousness. Based on this list, a positive marker can be identified that requires that all these capacities be present in a target system.^27^ This is called a *transition marker* because it marks the completion of an evolutionary transition from nonconscious to conscious animals.

According to Ginsburg and Jablonka, a transition marker for consciousness is *Unlimited Associative Learning (UAL)*. An animal with the capacity for UAL can learn about itself and the world in an extensive and open‐ended way, meaning that the number of possible associative links (i.e., learnable associations) is so large that the animal cannot learn all these links within its lifespan (Birch et al., 2020). UAL requires a set of features that are claimed to jointly suffice for consciousness (e.g., an evaluative system, unification of sensory inputs, and intentionality) so it is said to mark the presence of consciousness.

Godfrey‐Smith's approach argues that insight on the evolution of consciousness‐related features (e.g., sensory/evaluative complexity) can also give us insight into the evolution of consciousness itself.^28^ One of his views is as follows: We start by considering a tree that shows how animals are related to one another, then map onto that tree consciousness‐related features such as sensory and evaluative complexity. Afterward, this mapping is taken to enable inferences about the distribution of consciousness itself based on what view one takes on the relation between consciousness and consciousness‐related features. Godfrey‐Smith proposes three views one might take: gradualist, threshold, or latecomer. On the *gradualist* view, the gradient of complexity on the sensory or evaluative side maps onto the gradient on the consciousness side. This means that whenever the features evolve more complexity, so does consciousness itself. It does not necessarily imply that the complexity of consciousness varies on a single scale, because it can also mean that additional complexity introduces, or maps onto, additional *dimensions* of consciousness. On the *threshold* view, evaluative or sensory complexity needs to be sufficiently complex in order to be experienced; that is, animals with less than a certain level of sensory complexity have no sensory consciousness at all. In the human‐centered *latecomer* view, only *distinctive* kinds of cognitive processing, typically spelled out by contemporary neuroscientific theories of consciousness, bring about consciousness. The implications of how consciousness evolved, then, can be drawn from choosing one of the three options.

We take all three of these views seriously. However, none of them involves an evolution‐first approach. No matter which of these views we choose, in this methodological approach, evolutionary evidence only becomes relevant once we have independently decided on one of these three types of theories of consciousness. Moreover, the evolutionary evidence which is used directly concerns features such as sensory complexity. Inferences about the evolution of consciousness are then based on an independently arrived at theory of consciousness in conjunction with the evolution of cognitive capacities which are not directly related to consciousness, only in virtue of the prior, nonevolutionary, theory. So, the evolutionary evidence only plays a secondary role in that it is based on a prior theory of consciousness. A similar point holds with respect to the UAL framework. UAL as a transition maker is based on theories of consciousness, and only afterward are the implications for the distribution and, ultimately, the evolution question explored. Evolutionary considerations are not taken as central to the plausibility of UAL as a transition maker. The central evolutionary claims are merely the result of top‐down reasoning from independent accounts of consciousness; they do not themselves play an independent and foundational role in advancing our understanding of human or animal consciousness.^29^

To summarize Sections 2 through 4, the examples and general considerations we have adduced provide a reason to think that evolution‐first approaches to consciousness are not viable. Claims about the evolution of consciousness need to combine knowledge about animal evolution with detailed accounts of the distribution, nature, and psychological function of consciousness in present‐day animals (human and nonhuman), which need to be mostly based on experimental research. Moreover, investigations of the distribution and nature of consciousness are largely driven by such experimental evidence. So, if our views on consciousness’ distribution and nature change, this will revise our views on the evolution question—but evolution‐first approaches cannot provide independent evidence to help with this shift. In conjunction, this gives contemporary evidence a more foundational role. Nevertheless, evolutionary thinking can have an important role, even if it is not a foundational role, which we will now describe in more detail.

## 5 The many roles of evolution

Here is a nonexhaustive list of the ways that evolutionary considerations can still help consciousness research.^30^

First, as Birch and Andrews (2023) point out, we can infer that consciousness evolved convergently in two species, s1 and s2, if we know that s1 and s2 are both conscious but that their common ancestor was not conscious. If consciousness can be shown to have the same psychological function in both species, then this is tentative evidence that consciousness necessarily has that function, which can be strengthened by considering more species. Notice how this inference, which centers on evolutionary convergence, only makes sense from an evolutionary perspective.

Second, if there is evidence that one clade of animals is conscious, then phylogenetic knowledge can motivate the hypothesis that its nearest relative is a candidate for consciousness. Then, this knowledge can encourage close exploration of the similarities and differences in consciousness between close phylogenetic relatives, such as humans and chimpanzees or arthropods and velvet worms (i.e., if arthropods are found to be conscious, we should look for this in their sister group, the velvet worms (Campbell et al., 2011)). This example shows that evolutionary reasoning is not only relevant in the context of justification, but also in the context of discovery (i.e., generating new hypotheses).^31^ It can also shed light on which questions are pursuit‐worthy (Shaw, 2022).

Third, even more basic, if many species within a clade are conscious, this suggests that others within the same clade are too. The evidence that a species is conscious is particularly strong if there is credible evidence of consciousness in both its earlier and later branching relatives on the phylogenetic tree.

Fourth, evolutionary knowledge can further strengthen correlations between consciousness in different organisms and other traits they possess. For example, Mallatt, Blatt, Draguhn, Robinson, and Taiz (2021) argue that there is a strong correlation between having elaborate sensory organs—posited to encode sensory maps of animals’ surroundings and their own body—, having larger brains, having high mobility, and satisfying other criteria for consciousness. Such correlations can be strengthened by considering not only today's extant animals, but also evolutionary trends documented by the fossil record.

Fifth, as indicated earlier, fossils can provide evidence when they are combined with plausible, independently tested, accounts of consciousness. For example, Ma, Hou, Edgecombe, and Strausfeld (2012) and Strausfeld & Hirth (2024) report similarities in size and structure between the fossil brains of Cambrian arthropods and the brains of modern arthropods such as crabs and spiders, respectively. This suggests that, if modern arthropods are conscious, arthropod consciousness is more than 500 million years old. Feinberg and Mallatt (2016, chapter 6) discuss evidence from endocasts of fossil vertebrate skulls which points to an enlargement of brains when the first mammals evolved, supporting the hypothesis that capacities related to consciousness changed at this same time. In addition, fossils can reveal evidence of behavior (e.g., whether an animal was a predator and which organisms it consumed), which can lead to insights into the history of consciousness when it is combined with a plausible account of the behaviors that consciousness facilitates. Finally, the fossil record reveals evolutionary accelerations—such as the Cambrian evolution of animal diversity—which may have played an especially important role in the evolution of consciousness.

Again, note that these evolutionary considerations also count as evidence from a falsificationist perspective. For example, hypotheses about the evolution of consciousness (e.g., that consciousness significantly changed ∼200 mya) can be falsified in recourse to the fossil record (e.g., fossils indicating no significant brain or behavioral changes 200 mya), if fossil evidence is interpreted in the light of plausible independent accounts of consciousness.

## 6 Conclusion

In this paper, we have argued against evolution‐first approaches according to which reasoning about evolution has an independent and foundational role in assessing (not necessarily generating) hypotheses about consciousness. We made the case that such accounts struggle to provide compelling empirical evidence for their key claims, even given the assumption that consciousness has an evolutionary function. This raises the question how, exactly, evolutionary reasoning can inform consciousness science. On our view, evolutionary considerations play a more modest, secondary role, but are still a highly important tool which can enrich consciousness science in a variety of ways. If proponents of evolution‐first approaches to consciousness disagree, we encourage them to spell out how precisely, on their view, evolutionary considerations evidentially support detailed and ambitious accounts of consciousness.

## Notes

1 Characterizing consciousness this way is widely adopted in contemporary debates, by physicalists (Balog, 2012; Papineau, 2002), dualists (Chalmers, 1996; Yetter‐Chappell, 2022), and scientists (e.g., Ginsburg & Jablonka, 2019; Lau, 2011; Overgaard, 2018; Seth & Bayne, 2022), because it is taken to be neutral with respect to most controversial views, yet substantive enough to characterize a core and (potentially) empirically tractable explanandum of scientific—and philosophical—investigation (Frankish, 2021; Schwitzgebel, 2016).

2 This assumption makes our life harder. Our subsequent argument against evolution‐first approaches would be much more straightforward if this assumption were false. While this assumption is by no means uncontroversial (see, e.g., Robinson, Maley, & Piccinini, 2015), it is shared by most consciousness researchers (Francken et al., 2022, Fig. 2, question 11) and is presupposed by all researchers central to the discussion in this paper.

3 This terminological stipulation does not commit us to any controversial metaphysical view about functions, such as the teleological account of functions. It is compatible with, but not required by, our argument that talk of functions in science and philosophy needs to be explained in a metaphysically substantive way. Whatever is the correct metaphysical account of function talk generally will apply to our argument too.

4 To illustrate, if dualism is true, the target of explanation of scientific inquiry would then shift toward identifying the underlying physical/functional processes that are systematically associated with P‐consciousness (Chalmers, 2010), and it remains open which processes these are. Scientific inquiry, then, still contributes to our understanding of consciousness (Chalmers, 2010, p. 40), even if consciousness is not *entailed* by physical/functional processes (Chalmers, 1996), and even if scientific knowledge cannot *wholly* explain it (Levine, 1983).

5 Having said that, some of our assumptions may need to be reinterpreted depending on one's metaphysical views. For instance, if epiphenomenalism is true, the assumption made earlier that consciousness has an evolutionary function can be reinterpreted as the assumption that the physical/functional processes giving rise to consciousness have an evolutionary function (Black, 2021). Many epiphenomenalists endorse a view of this kind (see, e.g., Jackson (1982, p. 134)). Given that we are interested in what the most fruitful way is to identify the physical/functional processes *associated* with consciousness, none of this affects our arguments against the evolution‐first approach.

6 For example, theories of consciousness are often tested against experimental paradigms which rely on verbal reports of human subjects (and occasionally nonverbal reports of monkeys or apes) to examine conditions in which stimuli are consciously or unconsciously perceived (e.g., binocular rivalry (Blake & Logothetis, 2002), masking (Kouider & Dehaene, 2007), and dichotic listening (Brancucci & Tommasi, 2011) conditions), patients with brain damage (e.g., patients with blindsight (Weiskrantz, 1986)), or brain‐imaging of patients in conscious versus unconscious conditions (e.g., patients under anesthesia (Alkire & Miller, 2005)) (see Shevlin, 2021 for details). Moreover, attempts to go beyond introspective reports, such as no‐report paradigms, remain controversial (see, e.g., Block, 2019; Michel & Morales, 2020) and are still validated by, or should be combined with, subjective reports by humans (Overgaard & Fazekas, 2016). Alternative approaches, such as the iterative natural kind strategy (McKilliam, 2024), are also inclined to take insights on humans as their initial source of evidence (McKilliam, 2024, p. 9).

7 The distinction between theory‐heavy, theory‐light, and theory‐neutral is adopted from Birch (2022).

8 In Section 5, we will give multiple examples of this secondary role.

9 In saying that evidence is foundational, we only claim that it is foundationally relevant for testing and assessing hypotheses. We do not mean to imply that hypotheses can be derived logically from empirical evidence which is arguably an outdated view of how scientific theorizing works.

10 Hence, our argument against the evolution‐first approach is consistent with evolutionary considerations playing a (central) role in the context of discovery (see Section 5).

11 See McKilliam (2024, Section 2) for a thorough discussion on why theory testing in consciousness research is best understood as a gradual affair as opposed to depending on some *experimentum crucis*, a single experiment (or series of experiments) which is supposed to decisively rule between competing theories.

12 This is consistent with thinking that contemporary evidence on human consciousness is nevertheless especially well‐suited to explaining certain specific features of *human* consciousness (Veit, 2023b, p. 25).

13 Action needs to be understood broadly as including any kind of functional activity an agent can produce.

14 Veit's approach is *theory‐neutral* in so far that it does not presuppose a particular theory of human consciousness. However, his approach is unlike traditional theory‐neutral approaches that rely on structural or functional similarities between humans and animals.

15 See Section 4.

16 There are other objections, less revealing about the evolution‐first approach, than the ones we pursue. First, Dung and Newen (2023) argue that there are 10 consciousness dimensions, which would—at least—make Veit's argument incomplete. More foundationally, Birch et al.’s selection of dimensions is pragmatically informed (Birch, Schnell, & Clayton, 2020, p. 797), so it may not even be intended to correspond to ontological divisions of consciousness. Second, Veit infers from the (putative) fact that a dimension is not necessary for consciousness that it is an evolutionary latecomer. But this does not straightforwardly follow, since dimensions that are not necessary may nevertheless have co‐evolved during the dawn of consciousness, only to break down in special circumstances.

17 But we note that there are many different accounts of valence in the literature (e.g., Carruthers, 2018; Cutter & Tye, 2011; Barlassina & Hayward, 2019; Klein, 2015; Heathwood, 2007; Damasio & Carvalho, 2013; Ginsburg & Jablonka, 2019) and it seems that only Veit's preferred common currency account (Cabanac, 1992, 1996; Levy & Glimcher, 2012) ties it *directly* to PC.

18 This interpretation fits most naturally with Veit's overarching project of a Darwinian bottom‐up approach to consciousness. Another way to interpret the dynamics between PCT and evolutionary considerations is to simply antecedently assume that the PCT is correct, and subsequently assess how the PCT fits into an evolutionary story. However, this method stands and falls with how plausible the PCT antecedently is.

19 Spurrett (2023) makes a related point that trade‐off problems associated with PC can be accommodated by solutions that do not involve consciousness, or perhaps not even an evaluative system.

20 Insects, for instance, bees, are sometimes mentioned as sensory specialists. However, recent evidence suggests that bees have rich evaluative capacities (Gibbons et al., 2022; Gibbons, Versace, Crump, Baran, & Chittka, 2022), so that sensory specialists may be pure hypotheticals.

21 We make this assumption only for the sake of argument.

22 We should note that evidence of sensory‐specialists can also simply be based on evidence about the current distribution of consciousness‐related capacities (i.e., sensory and evaluative capacities), without any evolutionary reasoning.

23 By the same principle, the existence of evaluation‐specialists, organisms with rich evaluative, but poor sensory, capacities, would not constitute evidence in favor of the PCT, contrary to what Veit claims (2022b).

24 To emphasize, while these additional features provide a more substantive and contentful characterization of the evolution‐first approach, they are not strictly speaking *essential* to it.

25 Likewise, if evolutionary considerations only play a role in inspiring a hypothesis about consciousness, where the confirmation (or falsification) of this hypothesis is taken to be based on evidence derived from living animals (and perhaps even AI systems) (see, e.g., Stewart, 2022), then it also does not count as evolution‐first.

26 An independent issue with the evolutionary cognitive archeology approach is that it typically limits its focus on the evolution of *hominin* cognition (Wynn & Coolidge, 2016, 2022). This makes the approach, at least when taken in isolation and constrained to hominin cognition, less suitable to directly inform answers to questions about the origins, and distribution, of consciousness in general.

27 Not just *any* positive marker will do. Between two markers that both require all capacities, the one that is distributed more widely across nonhuman animals is preferred.

28 Godfrey‐Smith's overall approach to consciousness is subtle and we cannot do justice to it here. It involves merging many different chains of reasoning and pieces of evidence into an overall story of the evolution of mind. Instead, we discuss here some of his specific, direct claims about the evolution of consciousness.

29 It is worth noting that Feinberg and Mallatt's *Neurobiological Naturalism (NN)* also fits nicely with the top‐down view being discussed here (Feinberg & Mallatt, 2020; see also Mallatt, 2021). NN starts with theoretical reasoning (Mallatt, 2021, p. 10), identifying plausible but tentative/refutable premises about consciousness, and evidence‐gathering based on these premises in present‐day animals. Only afterward is the methodology applied to evolutionary questions (see, e.g., Mallatt, 2021, p. 11; Mallatt, Blatt, Draguhn, Robinson, & Taiz, 2021, p. 12).

30 We thank an anonymous reviewer for suggestions which have significantly contributed to this section.

31 We take it that evolutionary reasoning can aid hypothesis generation regardless of what the nature of this process is (i.e., whether it amounts to an analyzable reasoning process or something akin to an unanalyzable creative act).
